# Combined PET and MRI for the masses! At least for the cardiac ones

**DOI:** 10.1007/s12350-021-02881-7

**Published:** 2021-12-21

**Authors:** Christoph Rischpler, Robert Seifert

**Affiliations:** Department of Nuclear Medicine, University Hospital Essen, University of Duisburg-Essen, Essen, Germany

About a decade ago, the dream of many nuclear physicians, radiologists, and even engineers came true and the first fully integrated, commercially available PET/MRI device was launched, leading to a large body of work investigating the synergy of these two imaging modalities.^1^ It did not take long for promising results to be published in oncological^2^ and also neurological fields of application.^3^ In cardiology, on the other hand, the integrated analysis of PET and MRI was initially mainly employed in research projects, either in clinical studies^4,5^ or case reports,^6,7^ albeit relatively numerous. The first real convincing clinical application was published in this very journal: a study was able to show the benefit of hybrid FDG PET/MRI in myocarditis patients.^8^ Since then, other clinically significant cardiac applications of combined PET and MRI imaging have been published. One that is particularly worth highlighting is a study of sarcoidosis patients with the clinical question of cardiac involvement.^9,10^ Here, combined PET and MR imaging has demonstrated its added value primarily by showing greater accuracy with combined analyses. Another very significant population of patients in whom added value of PET/MRI has been suggested is patients with cardiac tumors. It is understandable that in these patients, the most accurate imaging is highly desirable to avoid the frequent need for diagnosis by biopsy. Although the majority of myocardial tumors, approximately three-quarters, are benign, misdiagnosis can of course have fatal consequences. On the other hand, due to the high complication rate of the procedure, an unnecessary biopsy in a benign myocardial mass can also result in high morbidity and rarely even lethality. While there was a pilot study back in 2015 that looked at the value of integrated analysis of PET and MRI in the study of cardiac masses, the major shortcoming of that study was the small case number of 20 patients.^11^ Another study that was limited to FDG PET/CT came up with only 24 patients as well.^12^ However, in the highly interesting study by Aghayev et al published in the current issue of the JNC, more than three times as many patients were examined, resulting in a cohort of 72 patients.^13^ Furthermore, histological correlation was present in the majority of patients. Last but not least, the patients were followed-up and thus high-risk features regarding outcome could be investigated. In the current study, MRI was shown to achieve high sensitivity and specificity in differentiating benign vs. malignant cardiac tumors if 4 of 9 MRI features were positive (AUC 0.91). Individual MRI features showed significantly lower accuracy, with, interestingly, mere lesion size showing the highest accuracy. The inclined reader (particularly nuclear medicine physicians) may take some pride in the fact that a single, simple feature of PET, namely an SUV_max_/blood pool ratio > 3, showed similar accuracy compared with the more complex MRI analysis (AUC 0.87). In the end, the integrated analysis of PET and MRI did not show a higher accuracy, but the authors could prove the added value in single complex cases. Outcome analysis also demonstrated that both MRI, by showing an infiltrative border, and PET, by showing focal extracardiac FDG uptake, may have prognostic significance. As promising as the results are, further questions remain unanswered: the PET analysis in the present study was relatively simplistic compared with the MRI analysis (maximal uptake in FDG PET vs 9 different features in MRI), so the question remains whether a more elaborate analysis of PET, e.g., using dynamic acquisition or additional parameters such as SUV_peak_, SUV_mean_, radiomics of PET, metabolic tumor volume, can further increase diagnostic accuracy (Table 1).

**Table 1:** Comparison of the diagnostic performance of FDG PET vs. MRI in cardiac masses

Study	No. of patients	FDG PET	MRI	FDG PET + MRI
Rahbar et al. ^12^	24	100%/86%	–	–
Nensa et al. ^11^	20	100%/92%	100%/92%	100%/100%
Aghayev et al. ^13^	72	85%/88%	98%/84%	98%/84%

Given percentage values are sensitivity/specificity

Nevertheless, the study by Aghayev et al has shown one thing: an integrated PET and MRI analysis represents a promising diagnostic tool of cardiac masses and has the potential to become the clinical standard for this question. We therefore advocate "PET and MRI for cardiac masses!".
